# Localization of 18S ribosomal genes in suckermouth armoured catfishes Loricariidae (Teleostei, Siluriformes) with discussion on the Ag-NOR evolution

**DOI:** 10.3897/CompCytogen.v6i3.2667

**Published:** 2012-09-26

**Authors:** Anderson Luis Alves, Rafael Splendore de Borba, Allan Pierre Bonetti Pozzobon, Claudio Oliveira, Mauro Nirchio, Angel Granado, Fausto Foresti

**Affiliations:** 1 Embrapa Pesca e Aquicultura (CNPASA), Palmas, Tocantins, Brazil Embrapa Pesca e Aquicultura (CNPASA), Palmas, Tocantins, Brazil; 2 Laboratório de Citogenética, Univ Estadual Paulista “Julio de Mesquita Filho” - UNESP, Rio Claro, São Paulo State, Brazil; 3 Departamento de Morfologia, Instituto de Biociências, Univ Estadual Paulista Julio de Mesquita Filho, Botucatu, São Paulo State, Brazil; 4 Instituto Limnológico, Universidad de Oriente, Caicara del Orinoco, Estado Bolívar, Venezuela

**Keywords:** Fish cytogenetics, fluorescent *in situ* hybridization, Loricariidae

## Abstract

The family Loricariidae with about 690 species divided into six subfamilies, is one of the world’s largest fish families. Cytogenetic studies conducted in the family showed that among 90 species analyzed the diploid number ranges from 2n=38 in *Ancistrus* sp. to 2n=96 in *Hemipsilichthys gobio* Luetken, 1874. In the present study, fluorescence *in situ* hybridization (FISH) was employed to determine the chromosomal localization of the 18S rDNA gene in four suckermouth armoured catfishes: *Kronichthys lacerta* (Nichols, 1919), *Pareiorhaphis splendens* (Bizerril, 1995), *Liposarcus multiradiatus* (Hancock, 1828) and *Hypostomus* prope *plecostomus* (Linnaeus, 1758). All species analyzed showed one chromosome pair with 18S rDNA sequences, as observed in the previous Ag-NORs analyses. The presence of size and numerical polymorphism was observed and discussed, with proposing a hypothesis of the Ag-NOR evolution in Loricariidae.

## Introduction

Fishes of the family Loricariidae are found in almost all South and Central America, from Costa Rica to Argentina and represent one of the world’s largest fish families, with about 690 species described and about 300 undescribed (Reis et al. 2003). Recently this family has been divided into five subfamilies: Neoplecostominae, Hypoptopomatinae, Loricariinae, Hypostominae and a basal subfamily Delturinae (Armbruster 2004). About 100 Loricariidae species have been karyotyped so far (Oliveira and Gosztonyi 2000, Alves et al. 2006). The diploid chromosome number ranges from 2n=36 in *Rineloricaria latirostris* Boulenger, 1899 (Giuliano-Caetano 1998) to 2n=96 in *Hemipsilichthys gobio* Luetken, 1874(Kavalco et al. 2004).

Ribosomal RNA genes are organized in fishes and in other groups as multiple copies of a repeated unit that consists of a transcribed zone with coding regions for the 18S, 5.8S and 28S rRNA genes, separated by internal and external transcribed spacers and surrounded by non-transcribed spacer sequences. The 18S rDNA gene probes by fluorescent *in situ* hybridization (FISH) have provided coincident markers with silver nitrate impregnation (AG-NOR) in nucleolar organizer region (Ag-NOR) in fish chromosomes (Paintner et al. 2002, Porto-Foresti et al. 2002, Fontana et al. 2003, Kavalco et al. 2005).

According to Foresti et al. (1981) the Ag-NORs with large size polymorphism and/or numeric polymorphism are frequent in Neotropical freshwater fishes. Thus, the detection of genes related with Ag-NORs is very important for the identification and characterization of these kinds of polymorphism (Wasko and Galetti Jr 2000). Ag-NORs size polymorphism is common in Loricariidae fishes, mainly in species with single Ag-NORs as in Hypoptopomatinae and Neoplecostominae, although, it can occur in species with multiple Ag-NORs as Hypostominae (Artoni and Bertollo 1996, Alves et al. 2003).

In the present study the localization of 18S rDNA genes was identified in four species for the first time. The results were compared to already published data on Ag-NOR, with the main objective of better understanding the changes involving ribosomal genes involved with Ag-NORs in Loricariidae fishes.

## Material and methods

Cytogenetic analyses were performed on chromosome preparations obtained from four species collected in rivers from Brazil and Venezuela: *Kronichthys lacerta* (Nichols 1919), *Pareiorhaphis splendens* (Bizerril 1995), *Liposarcus multiradiatus* (Hancock 1828) and *Hypostomus* prope *plecostomus* (Linnaeus 1758) (Table 1). The specimens were analyzed by taxonomists that provided the species identification. The fishes were deposited in the fish collection of Laboratório de Biologia e Genética de Peixes (LBP), UNESP, Botucatu, SP, Brazil and in the Laboratório de Ictiologia, Museu de Ciências e Tecnologia, PUCRS (MCP), Porto Alegre, Brazil.

**Table 1.** A summary of the cytogenetic data available on the family Loricariidae with chromosomal localization ribosomal genes. 2n= diploid number; M= metacentric; SM= submetacentric; ST= subtelocentric; A= acrocentric.

**Species**	**Locality**	**rDNA gene**	**2N**	**Karyotypic formulae**	**Reference**
**Neoplecostominae**					
*Kronichthys lacerta* (Nichols, 1919)	Marumbi River, Brazil	18S	54	20M, 20SM, 14ST	Present study
*Pareiorhaphis splendens* (Bizerril, 1995)	Marumbi River, Brazil	18S	54	20M, 20SM, 14ST	Present study
*Neoplecostomus microps* Steindachner, 1877	Paraiba do Sul River, Brazil	18S	54	24M, 20SM, 10ST	Kavalco et al. (2005)
**Delturinae**					
* *Hemipsilicthys gobio* Luetken, 1874	Paraiba do Sul River, Brazil	18S	96	16M, 08SM, 72A	Kavalco et al. (2005)
**Loricariinae**					
*Harttia loricarifomes* Steindachner, 1877	Paraiba do Sul River, Brazil	18S	56	16M, 22SM, 10ST, 8A	Kavalco et al. (2005)
**Hypostominae**					
*Liposarcus multiradiatus* (Hancock, 1828)	Orinoco River, Venezuela	18S	52	22M, 18SM, 12ST	Present study
*Hypostomus affinis* Steindachner, 1886	Paraiba do Sul River, Brazil	18S	66	14M, 14SM, 12ST, 26A	Kavalco et al. (2005)
*Hypostomus* prope *plecostomus* (Linnaeus, 1758)	Orinoco River, Venezuela	18S	68	12M, 16SM, 12ST, 24A	Present study

Chromosome preparations were obtained from kidney tissues using the technique described by Foresti et al. (1993) and were submitted to fluorescent *in situ* hybridization (FISH). Four probes employed in the Southern hybridizations techniques were used for FISH and they were labeled as follow: the double-strand probes (probes 1 and 4) were labeled by nick translation with biotin-14-dATP (Bionick labelling system-Gibco.BRL); the single-strand synthetic probes (probes 2 and 3) were labeled by random primer with biotin-14-dCTP (BioPrime DNA labeling system-Gibco.BRL). The metaphase chromosomes slides were incubated with RNAse (40 µg/ml) for 1.5 hour at 37°C. After the denaturation of chromosomal DNA in 70% formamide/2xSSC for 5 min at 70°C, 40µl of hybridization mixture (100ng of denatured probe, 50% formamide, 10mg/ml dextran sulfate, 2xSSC) was dropped on the slides and the hybridization was performed overnight at 37°C. Hybridization washes included 50% formamide in 2xSSC at 42°C and 2xSSC and 4xSSC at room temperature. Detection of hybridized probes was carried out with Avidin-FITC conjugate (Sigma) followed by two rounds of signal-amplification. After each step of amplification the slides were washed in a blocking buffer (1.26% NaHCO3, 0.018% sodium citrate, 0,0386% Triton/1% non-fat dried milk). Chromosomes were counterstained with Propidium Iodide, and the slides were mounted with Antifade (Vector).

## Results and discussion

The karyotypes of the four species analyzed have been previously described in Alves (2005), Alves et al. (2005) and Alves et al. (2006), the diploid number and karyotype formulae with morphological classification in metacentric (m), submetacentric (sm) and subtelocentric (st) are presented in the Table 1.

**Figure 1.**
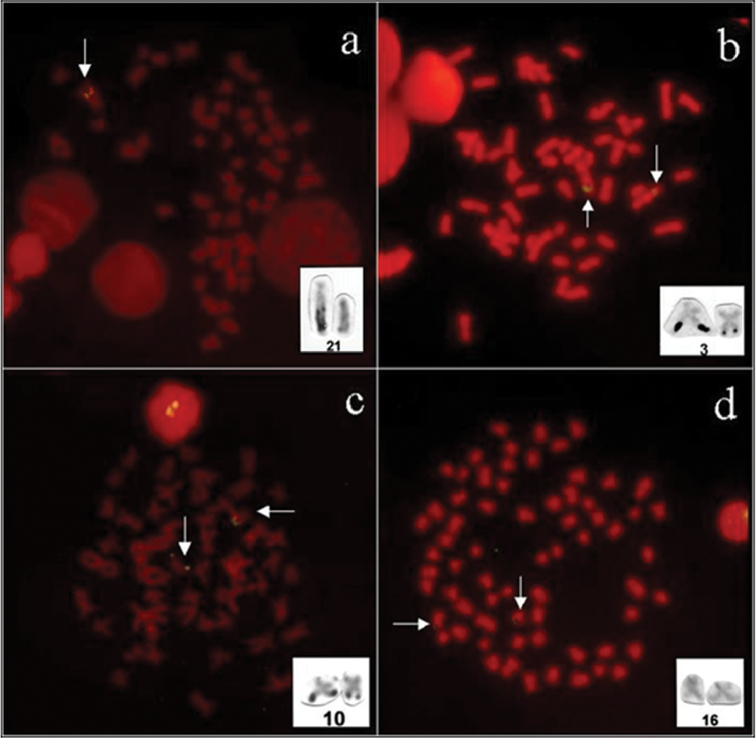
Fluorescent *in situ* hybridization with 18S rDNA probe in (**a**) *Kronichthys lacerta*, (**b**) *Pareiorhaphis splendens*, (**c**) *Liposarcus multiradiatus* and (**d**) *Hypostomus* prope *plecostomus*. Arrows indicate rDNA sites in the chromosomes pairs, Ag-NORs chromosomes are presented in the box.

The results showed that *Kronichthys lacerta* had only one signal of 18S rDNA in interstitial position in the long arm of the chromosome pair 21 (st) (Figure 1a), coinciding with a single Ag-NORs pattern presented by Alves et al. (2005). Beside the numerical polymorphism in this specie, the large size polymorphism of the 18S rDNA loci suggests a duplication of this gene in the active Ag-NOR chromosome.

*Pareiohaphis splendens* had two signal of 18S rDNA in interstitial position in the long arm of the chromosome pair 3 (m) (Figure 1b), coinciding with single Ag-NOR pattern presented by Alves et al. (2005). This species presented an evident variation in Ag-NORs size among homologous chromosomes that can be confirmed by the probe 18S rDNA. This structural polymorphism is common in the Loricariidae fishes (Artoni and Bertollo 1996, Alves et al. 2003, Alves et al. 2005).

In *Liposarcus multiradiatus* two signal of 18S rDNA in subterminal position in the long arm of the chromosome pair 10 (m) were detected (Figure 1c), coinciding with single Ag-NORs pattern presented by Alves et al. (2006). In this species a small size polymorphism of 18S rDNA occurs, although, the possible duplication or rearrangement events are not evident.

Weak signals of 18S rDNA were presented in the *Hypostomus* prope *plecostomus*: two signals were observed in the short arm of the chromosome pair 16 (st) (Figure 1d). These signals are coincident with single Ag-NORs presented in this specie by Alves (2005). Different of the others species analyzed here, *Hypostomus* prope *plecostomus* does not presented size polymorphism of 18S rDNA, suggesting a conserved status of this character in this species.

There are few studies related to the identification of Ag-NOR regions through the technique of hybridization with 18S rDNA probes in Loricariidae fishes, the most data available are on *Hypostomus*. The hybridization techniques with the fluorochromes DAPI and CMA_3_, were used to identified Ag-NOR regions of *Hypostomus nigromaculatus* (Schubart, 1964) (Rubert et al. 2008) and *Hypostomus* prope *wuchereri* (Günther, 1864) (Bitencourt et al. 2011). Artoni and Bertollo (1999) already used Mithramycin A (DAPI/MM) technique for observation of this region in *Hypostomus* sp., *Hypostomus* sp. B and *Hypostomus* sp. F. In a recent paper Mendes-Neto et al. (2011) identified the Ag-NOR regions in *Hypostomus regani* (Ihering, 1905) through the technique of hybridization with 18S rDNA probes, in all these works the species analysed showed single Ag-NOR in there chromosomes.

In conclusion, for Oliveira and Gosztonyi (2000) the condition of single Ag-NORs in subterminal position is the possible basal condition for the Siluriformes species, and variations of this character were considered derived. In the present study all species analyzed presented single Ag-NORs, suggesting the maintenance of this basal condition. The size polymorphisms observed in most species analyzed, suggests that these polymorphisms occurred independently of the species systematic position.

## References

[B1] Alves AL (2005) Análise da Evolução da Família Loricariidae (Telesotei, Siluriformes) Com Base em Caracteres Cromossômicos e de Seqüências de DNA. PhD Thesis, Botucatu (SP), Universidade Estadual Paulista.

[B2] Alves AL Oliveira C Foresti F (2003) Karyotype variability in eight species of the subfamilies Loricariinae and Ancistrinae (Teleostei, Siluriformes, Loricariidae). Caryologia 56: 57-63

[B3] Alves AL Oliveira C Foresti F (2005) Comparative cytogenetics analyses in eleven species of the subfamilies Neoplecostominae and Hypostominae (Siluriformes, Loricariidae). Genetica 124: 127-136.10.1007/s10709-004-7561-4 16134327 10.1007/s10709-004-7561-4

[B4] Alves AL Oliveira C Nirchio M Granado A Foresti F (2006) Karyotypic relationships among the tribes of Hypostominae (Siluriformes: Loricariidae) with description of XO sex chromosome system in a Neotropical fish species. Genetica 128: 1-9.10.1007/s10709-005-0715-1 17028935 10.1007/s10709-005-0715-1

[B5] Armbruster JW (2004) Phylogenetic relationships of the suckermouth armoured catfishes (Loricariidae) with emphasis on the Hypostominae and the Ancistrinae. Zoological Jounal Linnean Society 141: 1-80.10.1111/j.1096-3642.2004.00109.x

[B6] Artoni RF Bertollo LAC (1996) Cytogenetic studies on Hypostominae (Pisces, Siluriformes, Loricariidae). Considerations on karyotype evolution in the genus *Hypostomus*. Caryologia 49 (1): 81-90.10.1023/A:1003957719178

[B7] Artoni RF Betollo LAC (1999) Nature and distribution of constitutive heterochromatin in fishes, genus *Hypostomus* (Loricariidae). Genetica 106: 209-214 10897794 10.1023/a:1003957719178

[B8] Bitencourt JA Affonso PRAM Giuliano-Caetano L Dias AL (2011) Identification of distinct evolutionary units in allopatric populations of *Hypostomus* cf. *wuchereri* Günther, 1864 (Siluriformes: Loricariidae): karyotypic evidence. Neotropical Ichthyology 9 (2): 317-324.10.1590/S1679-62252011000200008

[B9] Fontana F Lanfredi M Congiu L Leis M Chicca M Rossi R (2003) Chromosomal mapping of 18S-28S and 5S rRNA genes by two-colour fluoirescent in situ hybridization in six sturgeon species. Genome 46: 473-477.10.1139/g03-007 12834065 10.1139/g03-007

[B10] Foresti F Almeida-Toledo LF Toledo-Filho SA (1981) Polymorphic nature of nucleolus organizer regions in fishes. Cytogenetic and Cell Genetic 31: 137-144.10.1159/000131639 10.1159/000131639 6173166

[B11] Foresti F Oliveira C Almeida-Toledo LF (1993) A method for chromosome preparations from large specimens of fishes using in vitro short treatment with colchicine. Experientia 49: 810-813.10.1007/BF01923555

[B12] Giuliano-Caetano L (1998) Polimorfismo cromossômico Robertsoniano em populações de *Rineloricaria latirostris* (Pisces, Loricariinae). PhD Thesis, São Carlos (SP), Departamento de Ciências Biológicas, Universidade Federal de São Carlos. 78 p.

[B13] Kavalco KF Pazza R Bertollo LAC Moreira-Filho O (2004) Heterochromatin characterization of four species of the family Loricariidae (Siluriformes). Hereditas 141: 1-6 10.1111/j.1601-5223.2004.01850.x 15703039

[B14] Kavalco KF Pazza R Bertollo LAC Moreira-Filho O (2005) Karyotypic diversity and evolution of Loricariidae (Pisces, Siluriformes). Heredity 94: 180-186.10.1038/sj.hdy.6800595 15562288 10.1038/sj.hdy.6800595

[B15] Mendes-Neto EO Vicari MR Artoni RF Moreira-Filho, O (2011) Description of karyotype in *Hypostomus regani* (Ihering, 1905) (Teleostei, Loricariidae) from the Piumhi river in Brazil with comments on karyotype variation found in *Hypostomus*. Comparative Cytogenetic 5 (2): 133-142.10.3897/compcytogen.v5i2.964 10.3897/compcytogen.v5i2.964 PMC3833738 24260625

[B16] Oliveira C Gosztonyi AE (2000) A cytogenetic study of *Diplomystes mesembrinus* (Teleostei, Siluriformes, Diplomystidae) with a discussion of chromosome evolution in siluriformes. Caryologia 53 (1): 31-37

[B17] Paintner-Marques TR Giuliano-Caetano L Dias AL (2002) Multiple NORs in *Bryconamericus* aff. *exodon* (Osteichthyes, Characidae, Tetragonopterinae). Hereditas 137: 107-112.10.1034/j.1601-5223.2002.01651.x 12627835 10.1034/j.1601-5223.2002.01651.x

[B18] Porto-Foresti F Oliveira C Tabata YA Rigolino MG Foresti F (2002) NORs inheritance analysis in crossing including indivisuals from two stocks of rainbow trout (*Oncorhynchus mykiss*). Hereditas 136: 227-230.10.1034/j.1601-5223.2002.1360308.x 12471670 10.1034/j.1601-5223.2002.1360308.x

[B19] Reis RE Kullander SO Ferraris Jr CJ (2003) Check list of the freshwater fishes of South America. Porto Alegre: Edipucrs, 729 pp.

[B20] Rubert M Zawadzki CH Giuliano-Caetano L (2008) Cytogenetic characterization of *Hypostomus nigromaculatus* (Siluriformes: Loricariidae). Neotropical Ichthyology 6 (1): 93-100.10.1590/S1679-62252008000100011

[B21] Wasko AP Galetti Jr M (2000) Mapping 18S ribosomal genes in fish of the genus *Brycon* (Characidae) by fluorescence in situ hybridization (FISH). Genetic and Molecular Biology 23 (1): 135-138.10.1590/S1415-47572000000100025

